# Peritonitis due to *Mycobacterium fortuitum* Following Gastric Banding

**DOI:** 10.4103/1319-3767.61239

**Published:** 2010-04

**Authors:** Fahad M. Al Majid

**Affiliations:** Department of Medicine, King Khalid University Hospital, College of Medicine, King Saud University, Riyadh, Kingdom of Saudi Arabia

**Keywords:** Hospital acquired, *Mycobacterium fortuitum*, peritonitis

## Abstract

*Mycobacterium fortuitum* is a rapid growing nontuberculous organism that has rarely been associated with peritonitis in an otherwise healthy host. We describe a patient who developed peritonitis due to the organism after gastric banding operation, which resolved after removal of the gastric band and institution of appropriate antibiotic therapy.

*Mycobacterium fortuitum* is a ubiquitous, rapidly growing organism that is readily cultured from soil, tap water, dust, and hospital environment.[1,2] When isolated from human sources (especially from the upper respiratory tract) usually reflects transient colonization rather than infection,[3] but it can cause skin and soft tissue infections following penetrating trauma and or even punch biopsy.[4,5] It has, however, been implicated in a number of postsurgical infections including infections of the sternum, mediastinum, and endocarditis, following heart surgery,[6] keratitis, and mastitis.[7] Peritonitis has been reported previously in many patients undergoing chronic peritoneal dialysis,[8] but has only been reported once before following abdominal surgery.[9]

We describe the first case of peritonitis due to *Mycobacterium fortuitum* after strict banding through laparoscopy for morbid obesity, in an immunocompetent woman.

## CASE REPORT

The patient was a 36-year-old female who presented to our institution with a two-month history of fever, chills, upper abdominal pain, and anorexia. A few days prior to the onset of presenting symptoms, she underwent gastric banding for morbid obesity in another facility. She had no other symptoms referable to other systems and was not on any medications.

Examination revealed an ill-looking morbidly obese lady with a body mass index (BMI) of 45. Vital signs were as follows: temperature 39°C, respiratory rate 26/minute, pulse 115/minute, and blood pressure 130/92 mmHg. Examination of the abdomen revealed a scar for the prior gastric banding, tenderness with rebound phenomenon especially on the left upper quadrant with clinical signs of ascites. Bowel sounds were normal. Examination of other systems was unremarkable.

**Figure 1:**
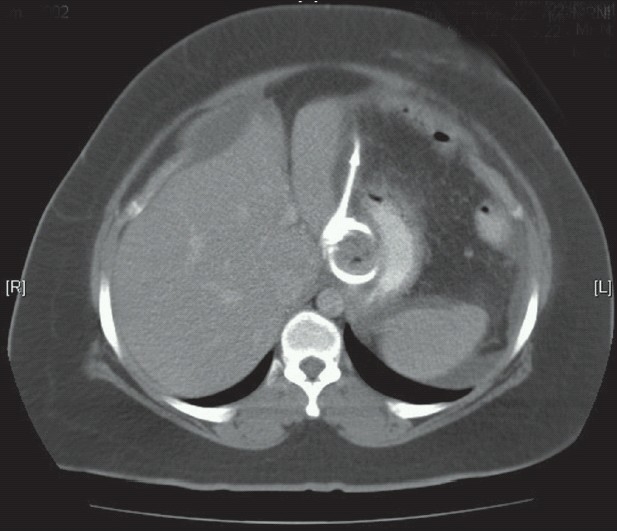
CT scan of the abdomen showing an enlarged liver with a gastric band *in-situ*

Initial investigations were as follows: white cell count 8.8×10^3^, hemoglobin 10.6 g/dl, platelets 412×10^3^, and erythrocyte sedimentation rate 112 mm/hr. Both chest radiograph and urinalysis were normal. Ultrasound scan and later, computed tomography (CT) scan of the abdomen confirmed the presence of free fluid in the peritoneal cavity, a gastric band *in situ* and an enlarged fatty liver [Figure 1]. Ascitic tap revealed an exudative fluid with total protein of 44 g/l, cell count of 450 cells/cuL comprising 80% of lymphocytes. Culture of ascitic fluid was performed by inoculating the specimen into a liquid culture media BACTIC MGIT960 (MGIT: mycobacterial growth indicator tube) a rapid growth was detected in six days. Fungal and other bacterial cultures revealed no growth.

Further identification was established and the diagnosis of *Mycobacterium fortuitum* complex was made. *Mycobacterium fortuitum* was differentiated from *Mycobacterium chelonei* by nitrate reduction and iron uptake, in addition to growth of the organism on MacConkey medium without crystal violet. In the specimen, antimicrobial susceptibility testing was done using broth microdilution and the result showed that the organism is resistant to rifampicin, ciprofloxacin and cefitriaxone, and sensitive to amikacin, gentamycin, clarithromycin, and tetracycline. Culture of gastric biopsy taken at endoscopy also grew the same organism. Gastrografin study did not demonstrate any leak into the peritoneal cavity. Blood cultures were sterile and tuberculin test was negative.

Hospital course: Patient was started on clarithromycin 500 mg orally twice daily, doxycycline 100 mg orally twice daily, and gentamycin 320 mg intravenously once daily. She remained febrile and symptomatic after a week of treatment. A decision to remove the gastric band was taken followed by rapid defervescence. Histology of the tissue around the band revealed foreign body type granuloma and culture grew the same organism with identical antiobiogram. Patient was discharged after three weeks of triple antibiotics. She was subsequently maintained on clarithromycin and doxycycline for a period of four months. She made complete and uneventful recovery.

## DISCUSSION

*Mycobacterium fortuitum* is a nontuberculous mycobacterium that is classified according to the Runyon grouping as rapidly growing mycobacteria (Runyon group IV). As the name implies, these types of organisms can be grown in culture and identified in less than seven days.[10] It was first described by Cruz who named it so because of its fortuitous isolation from the pus of a subcutaneous abscess.[11] It is an opportunist pathogen which ordinarily does not cause tuberculosis and occurs most often with abscess formation or indolent lung disease. Infection is often chronic, does not respond to conventional antituberculous treatment and occasionally remits spontaneously.

The organism is ubiquitous and is commonly found in the air, tap water, distilled water for dialysis and for preparing surgical solutions. It is, however, not a clear source of infection in the previous hospital where the patient had the operation and no other patients in the referring hospital were subsequently identified with a similar infection. The isolation of the organism in the gastric biopsy during endoscopy of our patient may suggest that the organism is available in the environment and might have contaminated the skin and or the equipment during surgery. The onset of symptoms shortly after surgery supports this hypothesis. Other possible cause is dissemination of the mycobacterium into the peritoneal cavity from gastrointestinal tract injury with leak during laparoscopy, although gastrografin study did not support this possibility. Nosocomial outbreak of infection by this organism has been reported in the surgical setting[12] and as respiratory tract colonization the latter being traced to a water line supplying showers in a ward.[13] Kobayashi *et al.* were able to isolate mycobacterial species from colonic contents during colonoscopy[14] confirming the normal colonization of the gastrointestinal tract by the organism.

According to Marks,[15] the organism is likely to be significant as an etiologic agent when isolated from the skin and subcutaneous tissue, and seldom so from other sources. Our ability to culture the organism in pure growth from both the ascitic fluid and the tissue around the gastric band, and the resolution on treatment, indicate that the organism is etiologically related to the clinical features of the patient.

*Mycobacterium fortuitum* is generally resistant to most antituberculous drugs. These drugs were, however, used widely for treatment until the 1990s when aminoglycosides alone and later in combination with macrolides (particularly clarithromycin), imipenem, and fluorinated quinolones were shown to be effective.[16,17] The national committee for the clinical laboratory standards (NCCLS) recommended broth microdilution technique to be the method for susceptibility testing of the Rapidly growing mycobacterium with minimum inhibitory concentration (MIC) determination and resistance breakpoint similar to those used for other bacterial species.[18] The antimicrobials used are selected bacterial agents and the recommendation for testing the rapidly growing mycobacteria (RGM) include clarithromycin (used as a class representative agent for the new macrolides), amikacin, cefoxitin, imipenem, doxycycline, ciprofloxacin, and sulfonamide. The current practice is to combine two or more of: amikacin, imipenem, cefoxitin, fluorinated quinolones, and macrolides in the treatment. The efficacy of this approach has been demonstrated in our case. No standard duration of therapy is reported but treatment lasting 4-6 months is not unusual. In patients with valve replacement and endocarditis due to this complex in which response was inadequate, removal of the infected prostheses led to resolution of infection.[19] Some patients in the same review, however, recovered without removal of infected valves. Another study has demonstrated higher case-fatality ratio in patients with foreign material, which may make it difficult to eradicate the organism once it is acquired.[9] The decision to remove the source of infection should therefore be taken on an individual case basis. Our patient demonstrated no response to therapy until the band was removed.

Death from *Mycobacterium fortuitum* infection is infrequent partly because most infections involve nonvital organs such as skin and soft tissues, and that the disease is generally self-limiting. Where death occurs, it is usually primarily due to the underlying disease with the *Mycobacterium fortuitum* infection playing a secondary role.

## CONCLUSION

There is a need to consider *Mycobacterium fortuitum* as a cause of postsurgical peritonitis and not just as a contaminant, do appropriate investigations and institute the right treatment. Current guidelines recommend susceptibility testing of all isolates, with use of empirical antibiotics suggested until drug sensitivities are known.
